# Supplementary material for: Metabolic syndrome and related factors in Cameroonian women under contraceptive use
Source: PLoS One. 2024 Nov 26;19(11):e0309054. doi: 10.1371/journal.pone.0309054 (PMC11594511; doi:10.1371/journal.pone.0309054)
Supplement: Attachment — Submitted filename: Response to Reviewers.docx [file pone.0309054.s002.docx]

**REBUTTAL LETTER**

**Journal requirements:**

1. When submitting your revision, we need you to address these additional requirements.

Please ensure that your manuscript meets PLOS ONE's style requirements, including those for file naming. The PLOS ONE style templates can be found at 
<https://journals.plos.org/plosone/s/file?id=wjVg/PLOSOne_formatting_sample_main_body.pdf> and <https://journals.plos.org/plosone/s/file?id=ba62/PLOSOne_formatting_sample_title_authors_affiliations.pdf>.

We have proofread the text and are reassured that it respects the style requested by PLOS ONE

2. Note from Emily Chenette, Editor in Chief of PLOS ONE, and Iain Hrynaszkiewicz, Director of Open Research Solutions at PLOS: Did you know that depositing data in a repository is associated with up to a 25% citation advantage (https://doi.org/10.1371/journal.pone.0230416). If you’ve not already done so, consider depositing your raw data in a repository to ensure your work is read, appreciated and cited by the largest possible audience. You’ll also earn an Accessible Data icon on your published paper if you deposit your data in any participating repository (<https://plos.org/open-science/open-data/#accessible-data>).

We're aware of the advantage of depositing our raw data in an accessible repository, but this study has collected a great quantity of data that is currently being exploited, so we don't want to make it available to the general public just yet. However, when this data is fully exploited, we will make it available to the general public in an online database.

3. We note that your Data Availability Statement is currently as follows: [All relevant data are within the manuscript and its Supporting Information files.]

Please confirm at this time whether or not your submission contains all raw data required to replicate the results of your study. Authors must share the “minimal data set” for their submission. PLOS defines the minimal data set to consist of the data required to replicate all study findings reported in the article, as well as related metadata and methods (https://journals.plos.org/plosone/s/data-availability#loc-minimal-data-set-definition).

For example, authors should submit the following data:

- The values behind the means, standard deviations and other measures reported;
- The values used to build graphs;
- The points extracted from images for analysis.

Authors do not need to submit their entire data set if only a portion of the data was used in the reported study.

If your submission does not contain these data, please either upload them as Supporting Information files or deposit them to a stable, public repository and provide us with the relevant URLs, DOIs, or accession numbers. For a list of recommended repositories, please see https://journals.plos.org/plosone/s/recommended-repositories.

If there are ethical or legal restrictions on sharing a de-identified data set, please explain them in detail (e.g., data contain potentially sensitive information, data are owned by a third-party organization, etc.) and who has imposed them (e.g., an ethics committee). Please also provide contact information for a data access committee, ethics committee, or other institutional body to which data requests may be sent. If data are owned by a third party, please indicate how others may request data access.

- The values used to plot the figures are indicated on each figure.

- Only a part of the collected data is used in this study, and therefore we cannot submit all the data in a single article.

- There is obviously highly sensitive information on the data collected which we must protect with regard to ethical clauses however, all relevant data are within the manuscript and its Supporting Information files.

4. Please include your full ethics statement in the ‘Methods’ section of your manuscript file. In your statement, please include the full name of the IRB or ethics committee who approved or waived your study, as well as whether or not you obtained informed written or verbal consent. If consent was waived for your study, please include this information in your statement as well.

- Done

**Reviewers' comments:
Reviewer's Responses to Questions**

**Comments to the Author**

1. Is the manuscript technically sound, and do the data support the conclusions?

The manuscript must describe a technically sound piece of scientific research with data that supports the conclusions. Experiments must have been conducted rigorously, with appropriate controls, replication, and sample sizes. The conclusions must be drawn appropriately based on the data presented.

Reviewer #1: No

Reviewer #2: Partly

I would like to reassure you that this study was rigorously carried out by a competent and very vigilant team. The results we obtained are repeatable, given that we were in the field with the nurses and operators to check and reassure ourselves of the seriousness with which the data was collected. However, we will revise the conclusion to bring it into line with the study's objectives.

2. Has the statistical analysis been performed appropriately and rigorously?

 Reviewer #1: No

Reviewer #2: No

As mentioned above, the data collected was a lot and therefore we used several scientific methods for the statistical analyses. We also believe that the statistical description is not very appropriate and rigorous. We have checked our basic data and proposed the right statistical analysis to fit this part of the study in order to remove your concern.

 3. Have the authors made all data underlying the findings in their manuscript fully available?

The [PLOS Data policy](http://www.plosone.org/static/policies.action#sharing) requires authors to make all data underlying the findings described in their manuscript fully available without restriction, with rare exception (please refer to the Data Availability Statement in the manuscript PDF file). The data should be provided as part of the manuscript or its supporting information, or deposited to a public repository. For example, in addition to summary statistics, the data points behind means, medians and variance measures should be available. If there are restrictions on publicly sharing data—e.g. participant privacy or use of data from a third party—those must be specified.

 Reviewer #1: Yes

Reviewer #2: Yes

As we have also said, the data from this study are being used for other scientific articles that are currently being written and submitted. For this reason, we cannot make available all the details of the raw data obtained from the field.

 4. Is the manuscript presented in an intelligible fashion and written in standard English?

PLOS ONE does not copyedit accepted manuscripts, so the language in submitted articles must be clear, correct, and unambiguous. Any typographical or grammatical errors should be corrected at revision, so please note any specific errors here.

 Reviewer #1: No

Reviewer #2: No

We have carefully proofread the manuscript to eliminate as many spelling and grammatical errors as possible. We now feel that the language is clear and well-written, making it easy to understand the themes addressed in this study.

 5. Review Comments to the Author

Please use the space provided to explain your answers to the questions above. You may also include additional comments for the author, including concerns about dual publication, research ethics, or publication ethics. (Please upload your review as an attachment if it exceeds 20,000 characters)

**Reviewer #1: Title: Metabolic syndrome and related factors in Cameroonian women under contraceptive use**

General comments.

The manuscript sought to address an important topical issue. However, there are many critical flaws and inconsistencies with regard to the objective, design, and results of the study. A comprehensive reconstruction of the article will be required. There are also lots of errors in grammar and sentence construction. A professional proofreading will be necessary.
Authors should also ensure the presentation of the manuscript and the references are in keeping with the journal requirements.

I've redesigned the study's objective, as well as the results, which are better presented and many spellings removed errors. I proofread the entire article and had it read by a professional in scientific language after re-structuring it. This helped to correct spelling and grammatical errors. The same goes for the bibliographical references, which were once again presented according to the journal's requirements.

Specific issues

Abstract
Page 2, lines 12-13: “Contraceptives can cause a myriad of secondary effects such as overweight, increase of blood pressure and triglycerides, and carbohydrate intolerance.” This is not true for all contraceptives. It is true for some types. Carbohydrate intolerance is the inability to digest certain carbohydrates due to a lack of certain enzymes. Glucose intolerance will be a more appropriate term here.

Corrected in accordance with your comments.

Lines 46 – 50: The use of 2012 data is very old for such an important statistic that is usually regularly updated. The same applies to reference 10 in lines 70-72, and some others.

These references have been updated

Lines 77-79: A cross-sectional study design cannot determine ‘effect’ (i.e. causality). It can at best determine associations.

I use the word “association”

Materials and methods

Study population: The description of the target and study population is unclear and confusing. If the target population were women on contraceptives, as stated by authors, the study population and sample can therefore not include women not on contraceptives.

We have corrected, bringing our sample size down to 231.

Lines 92-93: “Participants were 93 recruited by the principal investigator in the various health facilities…” The details of the sampling technique should be stated.

we've tried to provide more details

Lines 94-96: “We have contact 271 women, of whom 231 were on modern contraceptives, 28 were not using contraceptives, and 12 did not consent to the study”. This statement is confusing. For example, if 12 women did not consent, how come authors kept repeating in different aspects of the manuscript that 271 were INCLUDED in the study? Furthermore, based on what the authors stated to be the target population, there cannot be inclusion of 28 who did not meet that criteria.

I've already corrected the sample size to 231 instead of 271. But, 12 women refused to take part in our study and 28 were not using contraceptives.

How many respondents were in the study i.e. to whom the questionnaire was administered and had their anthropometrics data, lifestyle factors, and biochemical parameters collected?

231 (I've corrected taking your comment into account)

Pg 7, Lines 132 – 135: “To measure the WC, the tape was positioned at the natural waist or at the lower curvature located between the last rib and the iliac crest”. Please clarify the meaning of this statement, especially ‘natural waist’ and ‘lower curvature’.

To measure waist circumference, the subject stands comfortably, weight evenly distributed between both feet, feet slightly apart. The measurement is taken in a horizontal plane on the smallest part of the waist, equidistant between the bottom of the ribs and the pelvis bone.

Lines 141-142: “Elevated BP as a component of MetS was 142 defined as ≥ 130/80 mmHg”. This contradicts earlier stated criteria viz lines 19-20, and 64-65.

The most recent of the NCEP recommendations, the Adult Treatment Panel III (ATP III) guidelines concerning the blood pressure is 130/85 mmHg as consider in this study.

Lines 152-155: “Each blood sample was labelled with the participants’ number to avoid errors of recording. Blood glucose values were defined as normal ˂ 1.1 g/l; limit 1 – 1.25 g/l and diabetic ˃ 1.25 g/l. Raised TG was defined as TG level 0.4 – 1.4 g/l irrespective of gender and hypertriglyceridemia at ˃ 1.4 g/l. Whereas, low HDL-c level was defined as HDL-c ˃ 0.5 g/l in women and hypocholesterolemia at ˂ 0.5 g/l”. The statement is confusing. For example, what is the difference between ‘raised TG” and ‘hypertriglyceridemia’, what is ‘hypocholesterolemia’ etc.

Major error that we corrected. Each blood sample was labeled with the participant's number to avoid recording errors. Blood glucose values were defined as normal between 1 and 1.25 g/l and hyperglycemia ˃ 1.25 g/l. High TG was defined as TG between 0.4 and 1.4 g/l, regardless of gender, and hypertriglyceridemia ˃ 1.4 g/l. In contrast, low HDL-c was defined as HDL-c below 0.5 g/l in women.

Statistical analysis: Chi-square was used to analyse which variables? And Mann-Whitney for which type of variables?

The Chi-Square test was not used in this study. We apologize for this error. However, the non-parametric Mann-Whitney test was used as an alternative to the Student's t test, as the small amount of data was not normally distributed.

Results
Line 173: It is incorrect to say 271 were surveyed or that 271 were included, for reasons earlier highlighted. It is also not possible to assess the prevalence of contraceptive use because, according to the authors, the target population (hence inclusion criteria) was to consist only of those on contraceptives

Exactly, we have corrected and considered the exact sample size to present the results and calculate the prevalence of contraceptive use.

Lines 176-177: “The background characteristics of this study population are shown in Table 1”. Table 1 does not however contain the prevalence of each sub-category of the background characteristics.

Table 1, although a little cluttered, does contain the prevalence in precise detail. As an example, for each age group we have the prevalence for each determinant of the metabolic syndrome in the *"yes"* column. And for each determinant of the metabolic syndrome, the total prevalence is shown at the bottom of the table.

Page 10, line 181: What is the definition of ‘healthy’ and ‘unhealthy’ subjects

we've corrected that instead of "healthy" it's "in good health" and instead of "unhealthy" it's "not in good health".

Line 181-183: “. Out of two hundred and thirty-one women, 36.37% (95.04 ±6.38) were obese; 58.44% (85.62 ±4.20) were hypertensive (DBP) and 50.65% (0.45 ±0.05) had dyslipidemia due to HDL-c imbalance…”. What are the figures in brackets? Also, the prevalence of hypertension and dyslipidemia stated here are different from the ones in Table 1.

This is the comment in table 2, and these prevalence’s can be found in the Unhealthy (not in health) section at the left of the table, where we have the frequency column (n%) and the mean ± standard deviation column, which we have represented in brackets.

Lines 185-187: The meaning of the following statement is unclear: “The means (±SD) of the components of MetS including other related variables like overweight, systolic blood pressure, total cholesterol, LDL-c, and triglycerides values were demonstrated among the study population”

… were measured in the study population." It’s a mistake. We just wanted to make it clear that some other parameters not included in the components of metabolic syndrome were nevertheless measured.

Table 2: it is difficult to appreciate what the purpose of this table is and the interpretation of the information contained therein. There are several issues with it but here are some examples: what is the meaning of ‘healthy’ or ‘unhealthy’? The n(%) for each of the variables is referring to what? What is ‘glycemia’? The p-values are tests of significance for which statistical test? What is the contribution of the table to the objective of the article? Etc.

The aim of Table 2 is to present the percentage of people who are unhealthy (Not in good health) for each health-related variable correlated with metabolic syndrome. The word healthy (In good health) means apparently in good health, and unhealthy (Not in good health) indicates that the respondent has a measured or dosed health-related variable that is not normal. The n(%) refers to the size, i.e. the number of people, as well as the percentage, as shown in the legend below the table 2. Glycemia is the level of glucose in the blood. The p-values are tests of significance for Mann-Whitney. The table 2 shows the different variables linked to the parameters of the metabolic syndrome that contribute to its determination.

Lines 191-193: “The risk of MetS increases in women using injectable contraceptives (58.82%), which are the subjects with at least three components of metabolic syndrome. It is otherwise controllable in those using the Intra-uterine device (11.76%) and implant (29.42%)…”. Like many such statements in different aspects of the manuscript, some of which have been highlighted earlier, this statement is difficult to understand.

This result shows the prevalence of MetS according to the type of contraceptive used. MetS has been defined in our methodology. However, anyone with MetS has at least three of the five criteria that constitute MetS.

What is being shown in Figure 1 is also unclear!

Figure 1 shows the prevalence of MetS according to the type of contraceptive used, with prevalence indicated above each diagram.

It is pertinent to note that there is nothing in the results presented that showed an analysis of any association between the use of contraceptives and MetS, in line with the objective of the article. In any case, that will not be possible given that the target population was meant to include only those on contraceptives.

Indeed, the study was based on contraceptive users. Now, in the analysis of metabolic syndrome markers, we have identified different types of metabolic syndrome variable damage, suggesting that contraceptive use would be related to the occurrence of these pathologies, depending on the type of contraceptive used. We could therefore conclude from the data in the literature that contraceptives are likely to induce the appearance of numerous pathologies, including metabolic syndrome.

Discussion
The discussion is difficult to follow and has some specific issues, which are not limited to the following:
1. There are lots of discrepancies between the facts being discussed and the results earlier presented, for example, in lines 206-208: “Table 1 shows that most of these women were Christians (88.31%), 50.65% of them were 207 singles, only 27.27% were housewives, and more than half had acquired at least a secondary 208 education (53.25%)” There is no such information on table 1.

2. Another example in line 251: ‘However, in our study, the contraceptive method used had little impact on the determinants of MetS.” The is no data reflecting this in the results and, in any case, the study design cannot make such a determination.
3. The discussion around the ‘prevalence’ of the use of contraceptives becomes superfluous in view of the reasons earlier mentioned i.e. the design of the study cannot determine that.

1. To remove any ambiguity, we have included an additional table on the socio-demographic characteristics of the study population.

2. The sentence in line 251 has been revised.

3. We note this comment

Conclusion: Given the issues raised regarding the methods and results, the study cannot make the conclusions stated. Furthermore, there is no data presented on the analysis of dietary profile (line 278)

Exactly. We've revised the entire conclusion to make it more relevant for our study.

**Reviewer #2:**

Line 17: This number seems to be different from what you have in the results section. 271 vs. 231.
The exact sample size is 231

Line 22: Why are those who were not contraceptives and those who did not consent being reported as part of the total sample size. The study design was a cross- sectional one so how does these different categories fit in?

This has been corrected, and these two categories (those who were not contraceptives and those who did not consent) removed from the sample.

Line 27-28: Metabolic syndrome consist of a myriad of conditions and the confounders are quite a lot. How were the author able to eliminate those confounders. Women on contraceptives can be exposed to other conditions that can predispose them to metabolic syndrome? So, what were the authors trying to achieve by measuring metabolic syndrome in such women? Then again, the best approach will have been to use two arms of women i.e., women on contraceptives and those not on it. This should have been age-matched and sample matched. Then we could have made some inferences from the results. As it stands now, there are a lot of confounding factors that can lead to a bias in the results.

We missed this detail when setting up our protocol. We will take this into consideration in our future investigations. The various factors that would have caused MetS were not considered in our study. We focused on establishing the prevalence of MetS in these specific women using modern contraceptives. Although it's difficult to conclude that it was these contraceptive methods that induced MetS, we qualified this by suggesting that other factors could also have caused this metabolic disorder, even though the prevalence was relatively low.

Line 29: The focus of the work was to look metabolic syndrome in women on contraceptive. Now in the conclusion the authors seems to be silent on this. Is there a reason?

We've already entered this error

Line 32-33: How did the authors arrive at this conclusion? No part of the work looked at dietary pattern.
The study on eating habits is a part that will be the subject of another publication, so it was a confusion that we have corrected.

Line 77-78: How was this done? How was the effect of contraceptive on metabolic syndrome done in this study? And how were the other confounders controlled for?

We did not control for the factors that cause MetS. We based ourselves on the literature indicating the damage caused by contraceptive use to sample women using contraceptives and directly assess MetS in them. In a further study, we will compare the two groups to assess the effect of contraceptive use on the occurrence of MetS.

Line 81: In the abstract, it was stated that 231 women were on contraceptive. So where from this (271)? Why was a cross-sectional study used?

The exact sample size is 231

Line 95: So what happened to the 12 that did not consent? were they added or excluded from the study?

The twelve people who did not give their consent were excluded from the study.

Line 116: Did the patients fast before the sample was taken?

The blood was collected under fasting conditions, as described in the methodology in section 4.3.

Line 119-120: By what method was the glucose determined?

Fasting blood glucose samples were collected using an Accu Chek® Active blood glucose meter and test strips.

Line 149-150: This has already been described under line 118-119. Please delete

This is a repetition that we have deleted from lines 149-150.

Line 152-155: Glucose measurements are usually done in mmol/l or mg/dl. Please revise. Same for TG and HDL-c

We have already done it in mg/dl throughout the article

Line 159: Why was the Mann-Whitney test used?

The non-parametric Mann-Whitney test was used as an alternative to the Student's t test, as the small amount of data was not normally distributed.

Line 173: This sample size is misleading. Please revise.

Done. It’s 231

Line 173-177: The descriptions given here cannot be found in the table so why is reference made to the table 1?

These results can be found in the new Table 1 inserted in this article.

Line 178-179: Table 1 is very confusing because of the stratification that was done. Why were the stratifications like obese, diabetic etc done? This makes it difficult to understand the data presented. The authors could have just presented the general demography without any stratification for readers to appreciate the dynamics of the studied population. As it stands now it’s very difficult to make any meaning from the table. Please revise
Line 181: What was used to classify the subjects as either healthy or unhealthy?

The stratification was made taking in consideration the components of the MetS (obesity, diabetes, hypertension ..) which is central in this study. We revised line 181 (… in good health and not in good health).

Line 182: How come the authors are now using 231 in their data analysis? What happened to those not on contraceptives or did not consent?

I've already corrected the sample size to 231 instead of 271. But we remove the 12 women refused to take part in our study and 28 were not using contraceptives from the sample size

Line 184: Are these women not those classified as healthy?

Yes, these women are in good health

Line 188: What was the criteria for classifying the women as health and unhealthy in table 2? The main focus of the work is on contraceptive use and Metabolic syndrome. So, what is the essence of this stratification? Why was mean and median measured in table 2?

According to the components of the MetS in table 2 and according to the respective normal values of each component, we categorized the participants into two groups; those in good health and those not in good health. The calculate mean and median correspond for each component to a group of participants of either a good value or out of the norm.

The unit of measurement for the glucose and the lipid profile are not standard. Please use the WHO approved units of measurement. Ref Table 2.

We have already done it in mg/dl throughout the article

The discussion section needs to be looked at again. There seem to be no coherence in syncing the results with the discussion.

We've proofread and corrected the discussion section. We hope it's much more understandable now.

Line 201: why are the sample size still 271?

The exact sample size is 231

Line 212-218. Provide supporting references for the statements made.

Yes it’s stated to [16] [17] references

Line 228-235: Statements not so clear. Please revise.

We have done a complete proofreading to bring more precision to the statements made.

Line 238: Why is 36.37% being described as 'many'?

We notice it and replace many by almost

Line 253-258: The reasons given for why the injectable method presents with a higher metabolic syndrome is not backed by literature in the discussion section. What is the pathophysiology of the effect of injectables on metabolic syndrome?

Injectable affect carbohydrate metabolism through the activity of progestin and promotion of more storage of glycogen in the liver. The main mechanism of action is the inhibition of follicular development, ovulation. So, our study was not aimed to describe mechanism of action or pathophysiology of contraceptive cause MetS.

Line 278: Was there any analysis of dietary habits in relation to metabolic syndrome? The conclusion needs to be written again with focus on the major findings of the study.

Yes, in this study we did an analysis of dietary habits in relation to MetS, but this is not introduced in this article. We have therefore rewritten the conclusion according to your guidelines

 6. PLOS authors have the option to publish the peer review history of their article ([what does this mean?](https://journals.plos.org/plosone/s/editorial-and-peer-review-process#loc-peer-review-history)). If published, this will include your full peer review and any attached files.

If you choose “no”, your identity will remain anonymous but your review may still be made public.

**Do you want your identity to be public for this peer review?** For information about this choice, including consent withdrawal, please see our [Privacy Policy](https://www.plos.org/privacy-policy).

 Reviewer #1: No

Reviewer #2: No
